# ‘The lights are on, and the doors are always open’: a qualitative study to understand challenges underlying the need for emergency care in people experiencing homelessness in rural and coastal North East England

**DOI:** 10.1136/bmjph-2024-001468

**Published:** 2025-02-20

**Authors:** Steven A Thirkle, Emma A Adams, Jill Harland, Deepti A John, Eileen Kaner, Sheena E Ramsay

**Affiliations:** 1 Population Health Sciences Institute, Newcastle University Faculty of Medical Sciences, Newcastle upon Tyne, UK; 2 Hexham General Hospital, Northumbria Healthcare NHS Foundation Trust, Hexham, UK

**Keywords:** Public Health, Emergencies, Community Health

## Abstract

**Introduction:**

People experiencing homelessness have high rates of emergency care attendance compared with the general population. This study explores the factors underlying the need for emergency care services among people experiencing homelessness in rural and coastal areas of North East England.

**Methods:**

The study was conducted in Northumberland and North Tyneside (North East England). One-to-one semistructured interviews were conducted with people experiencing homelessness. Interviews and focus groups were undertaken with frontline staff from housing associations, police, ambulance services, emergency care, primary healthcare, mental health services and alcohol and drug recovery services. Discussions centred on emergency care experiences, reasons for access and underlying health and social needs.

**Results:**

Participants included 20 people experiencing homelessness (aged 18–56, 70% male) and 18 service professionals (aged 20–56, 56% female). Emergency care was often viewed by participants as an accessible safe place. Four key themes were found in this rural and coastal context: accessibility challenges due to limited public transport and geographic isolation; fragmented support exacerbated by widely dispersed services; service restrictions and limited alternatives having particular impact where options are few and prioritisation of immediate needs influenced by limited local resources.

**Conclusion:**

Challenges in accessing primary healthcare and social care, alongside varying levels of timely support and understanding of individual contexts, can contribute to the increased use of emergency care for people experiencing homelessness in rural and coastal areas. Integrating services with a focus on flexibility could be crucial for addressing the needs of these populations. This involves adapting to the unique circumstances of multiple deprived groups who lack access to community support.

**WHAT IS ALREADY KNOWN ON THIS TOPIC**

**WHAT THIS STUDY ADDS**

- This study integrates perspectives from people experiencing homelessness and providers in rural and coastal North East England, revealing how geographical isolation and service fragmentation create unique challenges for emergency care access and delivery.

**HOW THIS STUDY MIGHT AFFECT RESEARCH, PRACTICE AND POLICY**

- Findings support the need for locality-specific strategies and integrated care models that address the distinct challenges of homelessness in rural and coastal settings.

## Introduction

 Homelessness is increasing in the UK and also around the world, and as a result, more people experiencing homelessness are presenting at emergency care services.^1^ This trend is reflected in the North East of England, with statutory homelessness data showing increases in both study areas. Between 2019 and 2024, Northumberland saw initial homelessness assessments rise by 83% and households in temporary accommodation increase nearly eightfold. Similarly, North Tyneside experienced a 21% rise in assessments and a threefold increase in temporary accommodation use.^2^ In 2023, the escalating cost of living crisis in Great Britain has led to an alarming increase in homelessness, with over 300 000 households now affected.^3^ Homelessness, particularly, hidden forms of homelessness (eg, sofa surfing and temporary accommodation), is on the rise in rural and coastal areas of the UK due to a confluence of factors, including a severe shortage of social housing, inadequate transportation infrastructure and the precarious nature of local economies.^24^,^8^ People experiencing homelessness often have high levels of physical and mental health needs,^9^ which can lead to high use of emergency care services compared with other health and social care services.^10 11^ One key reason for this is the struggle to register with a general practitioner (GP), which is essential for accessing primary care since registration typically requires a fixed address.^12^ Furthermore, their health issues often arise from crises, which are not conducive to scheduled appointments.^13 14^ Factors that can increase reliance on emergency care include challenges accessing preventive care, logistical barriers, such as transportation and time constraints, experiences of stigma and discrimination and complex health needs that may complicate system navigation.^9 10 15 16^ Accessing non-emergency health and social care services can require a level of stability provided by a home,^17^ whereas emergency care services are immediate and more responsive to support during crises.^9^

People experiencing homelessness often find themselves in precarious conditions, which can perpetuate a persistent state of crisis.^18^ These conditions, shaped by both individual circumstances and environmental factors, such as harsh weather, lack of shelter and societal attitudes, heighten vulnerability and the urgency for care.^19^ Additionally, Levesque’s framework outlines that accessing health systems demands abilities—perceiving, seeking, reaching, paying and engaging^20^—that require resources often unattainable for the homeless due to instability, driving them towards emergency care.^21^ These vulnerabilities constitute some of the factors behind why people experiencing homelessness reach out to emergency care services in the first instance.^22^ A resource shift is required by people experiencing homelessness, from other duties, such as meeting basic needs, to active help seeking, which can present as a risk to recovery,^23^ as the homelessness experience demands attention to survival and not recovery in the long term.

Existing research often focuses on urban homelessness, leaving rural and coastal areas understudied. Recent research in urban areas of North East England, particularly in Gateshead and Newcastle upon Tyne, has provided valuable insights into healthcare access for homeless populations.^12 15^ Our study extends this regional understanding by specifically focusing on rural and coastal areas, which present distinct challenges from their urban counterparts. Accessing healthcare in rural and coastal areas is challenging due to the geographical dispersion of services and limited comprehensive provision, which is harder to justify in less densely populated areas.^4 24 25^ Emergency care presentations by people experiencing homelessness in rural and coastal areas are often related to co-occurring substance use and mental health and social issues.^26^ Little is known about pre-emergency care challenges for the homeless in non-urban areas in the UK, making it unclear how best to support them and prevent urgent presentations in busy hospital settings. This study aimed to identify and understand the key determinants driving emergency care use among people experiencing homelessness in Northumberland and North Tyneside. Understanding these determinants is crucial for developing early interventions and targeted prevention strategies, ultimately aiming to improve healthcare outcomes for this population.

## Methods

This study employed a qualitative, cross-sectional design, conducted between June 2022 and December 2022. The study aimed to understand experiences through semistructured interviews and focus groups with both people experiencing homelessness and service providers. The study is based in Northumberland and North Tyneside, which are large geographically diverse areas, including rural, coastal and urban areas, distributed across almost 2000 square miles in North East England. Northumberland is primarily a rural county, whereas North Tyneside comprises rural, coastal and suburban. Inclusion criteria for participants with lived experience were age 18 or older, current or recent (within the past 2 years) experience of homelessness in Northumberland or North Tyneside and ability to provide informed consent. For staff participants, inclusion criteria included current employment in a role involving direct work with people experiencing homelessness. Exclusion criteria included acute mental health crisis or intoxication at the time of interview. In this study, emergency care refers to hospital emergency departments, ambulance services, urgent care centres and minor injury units. The data also include references to experiences with non-emergency healthcare, such as general practitioners and outpatient services, to provide a comprehensive view of healthcare access among people experiencing homelessness in rural and coastal settings. This study follows the Consolidated Criteria for Reporting Qualitative Research guidelines (checklist submitted as a part of manuscript submission).

### Patient and public involvement

We gathered input from people with lived experience at different stages of our study. Prior to the start of recruitment and data collection, a pilot interview with a person experiencing homelessness was conducted in order to test and develop the interview questions. This initial interview informed revisions to our topic guide, ensuring that our questions were relevant and sensitive to the experiences of participants. We also presented early findings of the study with a lived experience group consisting of formerly homeless individuals, who actively participated in interpreting the findings and shaping the analysis. Their insights were invaluable in enhancing the validity of our conclusions. Furthermore, we gathered additional input from partner colleagues in practice and engaged with participants at larger community events to disseminate our findings and obtain further feedback and the results were presented to a lived experience group for their assistance with interpretation.

### Recruitment and data collection

The lead author conducted semistructured interviews with people experiencing homelessness, and interviews and focus groups with staff in health, housing and support sectors in Northumberland and North Tyneside. Using convenience and snowball sampling, 18 out of the 30 staff members contacted via email participated in the study. Individuals with lived experience of homelessness were recruited indirectly through service staff nominations, ultimately resulting in 20 participants. To reflect the diverse types of homelessness in rural and coastal regions, a broad definition of homelessness was used in recruitment, encompassing various forms, such as rough sleeping, those in temporary accommodations like hostels, individuals staying with friends or sofa surfing and those approaching local authorities for housing assistance.^27^ This definition, based on the Crisis typology, was particularly relevant for understanding homelessness in rural and coastal areas where hidden forms of homelessness are often more prevalent than visible rough sleeping. Staff were recruited to represent different levels and sectors. Participants received detailed study information, and audio or written consent was obtained. Most interviews were conducted in-person at service locations with safety protocols, except two phone interviews arranged by support workers. Safety protocols included conducting interviews in safe, semipublic locations, having a second researcher aware of interview times and locations, establishing check-in procedures and having clear protocols for terminating interviews if participants became distressed. Recruitment proceeded until reaching data sufficiency, indicating sufficient depth of insights. Participants with lived experience were compensated with a £25 voucher. Two semistructured topic guides were prepared: one for people with lived experience of homelessness and another for staff discussions. Interviews with people experiencing homelessness were around 20 min, while staff interviews and focus groups, blending in-person and online, were on average 50 min each. No participants dropped out or refused to take part. Study materials, including the consent form (online supplemental material 1), participant information sheet (online supplemental material 2) and topic guides for service users (online supplemental material 3) and service staff (online supplemental material 4), are available as supplementary files.

The National Health Service (NHS) Health Research Authority: National Research Ethics Service Committee in West Midlands Edgbaston provided ethical approval for this study on 4 May 2022 (Research Ethics Committee reference: 22/WM/0099).

### Data analysis

Interviews were digitally recorded, transcribed verbatim and checked for accuracy. Transcripts were imported into Nvivo V.1·6·1^28^ to support data management and analysis. For anonymity purposes, all identifiers (including participant’s and non-participant’s characteristics, such as name, gender and age) were removed. The interview transcripts and notes collected by the researcher during interviews were analysed using the framework method^29^ to develop themes. This systematic approach involves familiarisation with the data, coding, developing and applying an analytical framework, and interpreting the data through charting and mapping. Initial themes were established based on the research questions and literature review. The data’s richness and diversity allowed for a wide exploration of content, leading to a nuanced understanding of the themes. The research team familiarised themselves with the interviews by listening and reading the transcripts multiple times. Each interview was coded line-by-line using the four main topic questions as parent codes, under which relevant child codes were sorted. The four main topic codes that served as parent codes were emergency care presentation, health issues, social issues and trauma. Patterns and initial themes were identified during this coding process. The main themes developed when the codes, initial themes, quotes and explanations were collectively analysed. Theme development and verification involved regular team meetings among SAT, EAA and SER where codes and themes were discussed until consensus was reached, with any disagreements resolved through discussion. The final codebook is available as online supplemental material 5.

## Results

**Figure 1:**
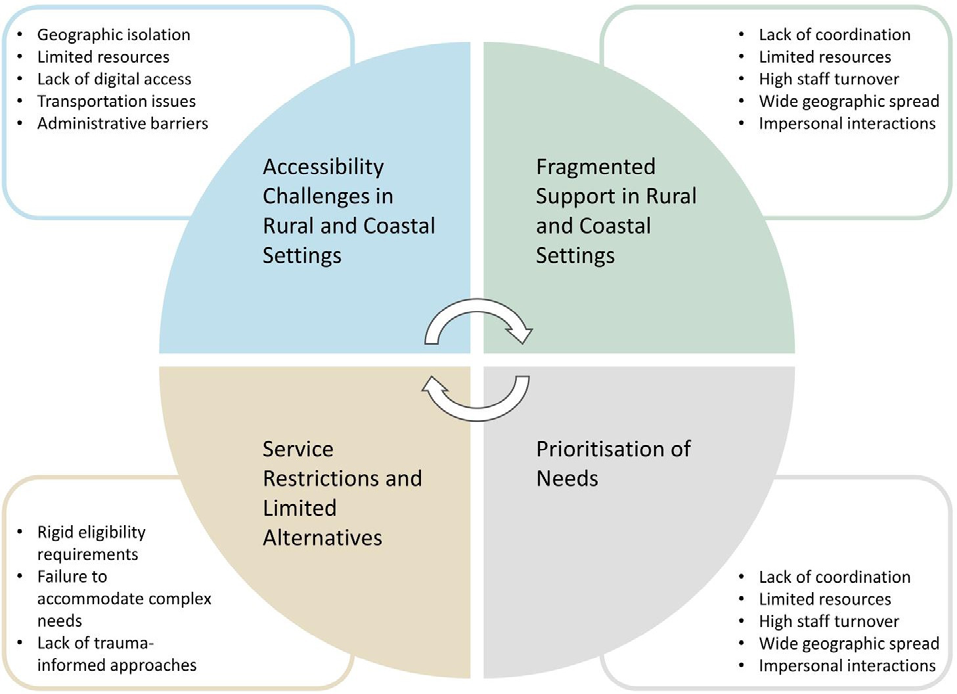
Determinants influencing emergency care use among people experiencing homelessness in rural and coastal settings.

A total of 38 participants were recruited for the study. Staff participants (n=18) represented diverse sectors, including emergency services (paramedics and police), healthcare (NHS Trust staff, GP), housing support and substance recovery services. Ages ranged from 20 to 56 years (mean 41·3, SD 10·2), and they were mostly female. Among people with lived experience (n=20), all had experienced rough sleeping, with current housing situations, including supported accommodation, staying with family/friends and hostel/hotel accommodation. Ages ranged from 18 to 56 years (mean 38·4, SD 9·5), and they were mostly male. The majority of participants were White British, with one participant identifying as non-White. There are four main themes described below and illustrated in figure 1.

### Theme 1: accessibility challenges in rural and coastal settings

Both people experiencing homelessness and service providers highlighted the significant impact of geographic isolation on accessing health and social care services in rural and coastal areas. In the rural and coastal areas of Northumberland and North Tyneside, accessing health and social care services for people experiencing homelessness was challenging due to geographic isolation, limited resources and a lack of digital access. This invisibility of the most vulnerable, coupled with assumptions of universal internet access and tech proficiency, results in services that were not adequately tailored to their needs.

‘A major issue is the invisibility of those most in need; services assume universal internet access, tech savviness and a stable address, neglecting those without—leading to a service not geared towards those who need it the most, causing many to fall through the cracks’. (Staff, Focus groups 22–25)

Many people experiencing homelessness participants found seeking assistance daunting and time-consuming, often encountering bureaucratic processes that could cause significant delays. These processes, compounded by limited and infrequent public transport, made emergency care the default option for those forced to travel long distances to urban centres.

‘But I can understand why… people go to emergency services when they are homeless because they have run out of options. It is a place with a light on, and the doors are always open, a bit of warmth, etc’. (Lived Experience, Interview 8)

Participants frequently mentioned systemic healthcare delays, including long waiting lists and limited appointment, which exacerbated their reliance on emergency care. This reliance was particularly pronounced in rural areas where limited transportation options further restricted access to scheduled healthcare services.

‘For years and years and years, I was on waiting lists, referral forms, calling up because the person who is ill is the person who has to ask for help, which is a bit hard because somebody who struggles with that, does not particularly feel like that they want help, might not even feel receptive or willing to be helped’. (Lived Experience, Interview 32)

Staff participants described the unique challenges of navigating rural health systems without a stable address, noting how mismatches between postcodes and GP locations could complicate access to entitled services

‘I think equally some people without a fixed address often do not really have a GP or do not have a relationship with a GP. Some services are commissioned by postcode and others are not and that does not always tie in because your GP surgery could be over the border, what we call the border. So, I think again, that could be confusing and complicated…’. (Staff, Focus groups 19 and 20)

Participants highlighted that individuals with learning differences and physical or mental disabilities often face significant obstacles in obtaining support, as the lack of specialised services and stringent eligibility criteria leave many in need without adequate assistance.

‘The council really let me down… I have got quite a few mental health issues… and they said I was not vulnerable’. (Lived Experience, Interview 15)

When formal support is denied despite mental health needs, people experiencing homelessness participants noted that people may be forced to rely on informal and potentially unsafe sources of support due to the limited availability of specialised services in these areas.

‘I was having a couple of drinks with them, and they were taking liberties on me because I could not walk’. (Lived Experience, Interview 33)

While accessibility challenges form the foundation of difficulties faced by people experiencing homelessness in rural and coastal areas, the fragmented nature of support services further compounds these issues.

### Theme 2: fragmented support in rural and coastal settings

The fragmentation of support services creates distinct challenges in rural and coastal settings, where geographic dispersion of services compounds coordination difficulties. Unlike urban areas where services might be colocated, rural services often operate across vast distances, making interagency communication and coordination particularly challenging. A person experiencing homelessness might need to travel several hours by infrequent public transport to access different services that, in urban areas, would be within walking distance of each other. Staff participants observed that effective communication among services is hampered by limited resources, systemic fragmentation, high staff turnover and the wide geographic spread of services. These challenges make it difficult for providers to maintain consistent communication and coordinate care.

‘When we refer to another agency… no one else sees it as urgent’. (Staff, Focus Groups 12 and 13).

This reflects the acute fragmentation of care delivery, particularly noticeable in rural and coastal settings.

People experiencing homelessness participants reported that service provision often overlooks individual circumstances and trauma histories, which can lead to ineffective or even harmful interactions. Limited funding, especially common in rural and coastal areas, further restricts the ability to address personal needs adequately.

‘Male staff coming to my door was bad… because of what I had been through’. (Lived Experience, Interview 28)

This oversight drives people experiencing homelessness towards emergency care services, emphasising the necessity for trauma-informed and sensitive approaches in care, especially in rural and coastal communities with limited specialised services.

Service interactions were often described as impersonal and bureaucratic, with many participants reporting feelings of dehumanisation and frustration, especially as these areas have limited other options.

‘You get triaged… there is no human in that at all’. (Staff, Interview 37)

The impersonal nature of these interactions can be particularly detrimental to individuals who are already vulnerable and marginalised, exacerbating their sense of isolation and helplessness. The fragmentation of services is closely linked to service restrictions and limited alternatives, which create additional barriers for those seeking support.

### Theme 3: service restrictions and limited alternatives

In rural and coastal areas, where alternative services are often distant or unavailable, service restrictions create particularly significant barriers to accessing healthcare. Unlike urban areas where multiple services operate within close proximity, exclusion from one service in rural and coastal areas can mean the next available option is hours away or unreachable by public transport. When people are excluded from services due to not meeting criteria or damaging relationships with providers, the impact is especially severe as there may be no other local options available. Staff participants highlighted that the challenge of managing complex needs often results in evictions from supported accommodation, reflecting a systemic failure to provide comprehensive, integrated care in areas with limited resources.

‘*If they are not receiving that support from someone else, something will happen in the house or they will be that out of control that ultimately what we have got to do is we have got to evict them because they are a risk to other people in the house’. (Staff, Focus Groups 12 and 13*)

Despite staff members fostering understanding and empathy through close community ties, frustration arises due to the constraints of available services. The impact of ‘burning bridges’ with services takes on heightened significance in rural and coastal settings, where alternative services are often non-existent within reasonable travelling distance.

‘I do feel we try and do whatever we can but there is a lot of people who have burnt their bridges with their behaviour. And that is quite difficult when you have got nowhere else to send them because nobody wants to take them back because of their behaviour before’. (Staff, Interview 18)

These limitations hinder staff’s effectiveness in meeting complex needs, highlighting the critical need for expanded and adaptable service provisions.

Participants highlighted that the exclusion of individuals who use substances from accessing mental health services intensifies the marginalisation of the most vulnerable, perpetuating recurring hospital visits without addressing the root causes of substance use.

‘Since I was 11 years old, I just wanted to have a drink, even though you go to hospital and they are telling you, no stop, I have been told about 10 or 12 times that I have got so many years to live, to pack in drinking but as soon as I am out of hospital, I am straight back on it again’. (Lived experience, Interview 30)

Staff participants expressed deep concern over the existing policy’s impact on individuals’ ability to recover and receive appropriate care.

‘We have got someone waiting for therapy… who is extremely unwell. I am frightened… she is so poorly. Without the intense support, I cannot see her stopping her alcohol use’. (Staff, Focus groups 22–25)

Participants highlighted that an exclusionary approach exacerbates health inequalities and perpetuates cycles of illness and substance misuse. Many people experiencing homelessness participants reported feeling ignored or dismissed by healthcare providers, eroding trust that discourages them from seeking help.

‘If you are not a drug addict… we cannot help you, go away’. (Lived Experience, Interview 16)

This reflects a broader issue of stigma and misunderstanding within healthcare systems, particularly impactful in rural and coastal areas with limited healthcare options less equipped to deal with diverse and complex cases. These service restrictions and limited alternatives, combined with the challenges of accessibility and fragmented support, often lead individuals to prioritise immediate survival needs over long-term health concerns.

### Theme 4: prioritisation of needs

In rural and coastal settings, people experiencing homelessness grapple with the necessity to prioritise basic needs over other health and social needs, influenced by the unique difficulties present in these environments.

Participants indicated that the urgency to meet immediate basic needs often drives individuals towards emergency care services, which are perceived as safe havens providing food and shelter.

‘Sometimes… it is somewhere that they can go where they can get fed’. (Staff, Focus Groups 6 and 7)

Participants noted that limited safe spaces and anonymity in less populated areas heighten dependency on these services, which can lead to vulnerabilities and often resulting in substance use as a coping mechanism.

‘…my housing is unstable because of who I am… I need to make sure that I am in a safe environment. I cannot function in situations and end up either turning to have a little drink to take the edge off, have a little smoke…’. (Lived experience, Interview 37)

Participants expressed that the struggle with self-care and managing daily life hinders addressing wider health and social needs.

‘When you are homeless… You are not taking care of yourself’. (Lived Experience, Interview 5)

Participants highlighted that, while emergency services provide temporary respite, the transition back to the external environment was challenging due to a dearth of supportive resources.

‘I get fed in hospital. There is a roof over my head. You get TV. You have got no worries. Whereas outside, you have got to pay your bills …it is so hard’. (Lived experience, Interview 36)

Participants conveyed that the prospect of recovery is overwhelming for people experiencing homelessness, especially in isolated areas where change can trigger anxiety and depression.

‘That side of it as well getting out and making that change just gives you anxiety’. (Lived Experience, Interview 4)

Both staff and people with experience of homelessness reflected on the constant pressure to meet immediate survival needs in rural and coastal settings and how it often overshadows longer term health considerations, creating a cycle that is difficult to break without comprehensive support.

## Discussion

People experiencing homelessness in rural and coastal areas could end up relying on emergency care services for their needs. While studies in urban areas of North East England have identified similar patterns of emergency care use,^12 15^ our study in rural and coastal areas of Northumberland and North Tyneside reveals how geographic isolation and limited transport options create additional barriers to accessing basic services. Our findings demonstrate a complex interplay among accessibility challenges, fragmented support, service restrictions and limited alternatives and the prioritisation of immediate needs. These factors are deeply interconnected, with geographic isolation exacerbating service fragmentation, which, in turn, forces individuals to prioritise survival over long-term health needs. The scarcity of community support services exacerbates the reliance on emergency care. The perspectives of both people experiencing homelessness and staff from service providers converge to highlight a system struggling to meet complex needs in challenging geographic and resource-limited contexts.

Lower reported levels of homelessness in rural and coastal areas result in fewer services being available when compared with urban settings.^4 6^ This resource and accessibility disparity risks the recovery of people experiencing homelessness, emphasising the need for a comprehensive care approach to address their complex needs.^21^ Additionally, the health and social care services in these areas are hindered by waiting lists, stringent entry requirements and a lack of immediate interventions for non-urgent conditions.^24^ These systemic barriers can further complicate access to care for people experiencing homelessness who need immediate assistance. Their negative experiences highlight the need for flexible systems, better service coordination and trauma-informed care. Emergency care services often become the primary recourse for people experiencing homelessness in rural and coastal areas.^9^

In rural and coastal settings, meeting the multiple and complex needs of people experiencing homelessness is notably challenging. While service scarcity affects homeless populations generally, previous research in rural areas has shown that geographic isolation creates unique patterns of service use.^25 30^ Our findings extend this understanding by demonstrating how the combination of distance and limited service availability creates particular barriers to recovery in rural and coastal settings. Staff awareness of other services can be more challenging to maintain in less densely populated areas, which may affect the provision of holistic care. Chaotic lifestyles of homelessness impede successful self-management, as highlighted in recent National Institute for Health and Care Excellence (NICE) guidelines.^31^ Further evidenced in a recent study of self-care perspectives among people experiencing homelessness, which found that people experiencing homelessness often prioritise immediate survival needs over health management.^32^ Our findings extend this understanding by showing how rural and coastal settings amplify these self-management challenges through geographic isolation and limited service availability. Individuals often endure their conditions rather than seek help due to the complexity of managing their needs and negative past experiences accessing help from services.^33^

Previous research has documented how complex service navigation can lead to self-help strategies.^1634^,^36^ Our findings demonstrate how rural and coastal geography intensifies these challenges, creating distinct patterns of substance use and help-seeking behaviour. These patterns align with studies conducted in other resource-limited settings, including urban areas during COVID-19,^15^ street homelessness^18^ and among those managing chronic conditions,^37^ though our findings highlight the additional impact of geographic isolation. This siloed approach is especially problematic in rural and coastal areas, where limited resources and specialised services amplify challenges faced by people experiencing homelessness. The limited support services in rural and coastal areas often mandate sobriety or substance use treatment before individuals can access other assistance, creating significant barriers seen similarly in countries like the USA.^38^ Substance use is frequently both a cause and a consequence of homelessness, reflecting complex interplay in these environments.^39^

The need for responsive homeless services has been widely documented.^18 40^ Constrained by limited resources, these services adopt exclusionary practices rather than inclusive ones. This not only heightens the risks associated with homelessness but also prolongs the duration. Our findings suggest that rural and coastal areas require specifically tailored approaches that account for geographic dispersion and limited resource availability.

Research shows that people experiencing homelessness have higher rates of childhood adversity and fewer positive role models compared with the general population.^41^ These early life experiences, combined with limited service capacity in rural and coastal areas, highlight the need for services that emphasise peer support and coordination.^21 42^ People in these situations prioritise immediate needs, leaving little room to learn skills for navigating out of their circumstances, echoing similar research on investigating meeting the needs of people experiencing homelessness attending emergency departments.^19 37^ People experiencing homelessness and staff highlighted the significant impact of past traumas on current situations, with services in rural and coastal areas often ill-equipped to address these issues sensitively, forcing individuals to relive the trauma and fostering distrust.^15^ The establishment of trust between people experiencing homelessness and providers is crucial for support access,^20^ and integrated care models, alongside shared assessments, could mitigate retraumatisation and address service fragmentation.^43^ The effectiveness of Finland’s interagency approach offers valuable insights for improving care in these settings.^44^ Evidence from discharge intervention studies has shown that coordinated transitions from emergency care can significantly improve postdischarge healthcare engagement, reduce hospital readmissions and increase treatment completion rates for people experiencing homelessness.^45^ These findings are particularly relevant in rural and coastal settings where geographic isolation can complicate discharge planning and follow-up care. Successful discharge planning in these settings requires consideration of transportation barriers, service availability and coordination across dispersed providers.^46^

Many of our findings reflect challenges documented in studies of homeless populations, such as prioritisation of immediate needs and impacts of trauma, suggesting that these are fundamental aspects of the homelessness experience. However, our study adds how rural and coastal settings could amplify these challenges through geographic isolation and limited service availability. In rural and coastal areas like Northumberland and North Tyneside, poor service coordination and scarcity hinder the addressing of complex needs. This further highlights the urgent need for integrated, trauma-informed care approaches. Our findings on fragmented support in rural and coastal settings align with challenges identified by both the Pathway model^17^ and the Horizon-funded CANCERLESS project.^14^ These frameworks emphasise the importance of coordinated care pathways and navigation support, approaches that could be particularly valuable in addressing the geographic and service coordination challenges this study identified. The success of these models in urban settings suggests potential adaptations for rural and coastal contexts, particularly in addressing the isolation and service fragmentation this study observed.

### Strengths and limitations

Through collaborative efforts between the research team and several services supporting people experiencing homelessness in rural and coastal parts of Northumberland and North Tyneside, this study successfully engaged a typically under-represented population in research. The qualitative approach allowed for rich, detailed exploration of participants' experiences and perspectives, providing insights that might not have emerged through quantitative methods. It strengthened its scope by including people experiencing homelessness with diverse experiences, spanning from emergency housing to independent living and substance support services. The inclusion of perspectives from both service provider and people experiencing homelessness enhanced the comprehensiveness of our findings. However, several limitations should be noted. While our study included services and participants from rural areas, the vast geographic spread of Northumberland meant some remote areas remained unreachable due to lack of service provision. Many people experiencing homelessness participants had originated from rural towns and villages but had been forced to relocate to more built-up areas to access support and accommodation, as these services did not exist in their home communities. This reflects a wider systemic issue in rural homelessness where service centralisation affects access patterns. Participants were recruited through services, potentially missing those completely disconnected from support systems. Additionally, as with all qualitative research, findings may not be generalisable to other rural and coastal settings, though they provide valuable insights into other rural and coastal areas experiencing similar challenges. Many of our findings, particularly around prioritisation of needs and experiences of trauma, mirror those seen in urban homeless populations, reflecting the shared challenges faced by people experiencing homelessness regardless of location, though these challenges are often amplified by rural and coastal geography. While we engaged individuals with lived experience during the pilot phase and on interpretation of the results, lived experience involvement was not sustained throughout the entire research process. Future research would benefit from a more integrated approach, ensuring that individuals with lived experience are actively involved at every stage, from study design to data interpretation. This would enhance the relevance and applicability of findings. The study participants may not be representative of the wider homeless population. This limits the generalisability of our results.^47^

### Implications for practice and future research

The study makes recommendations for policymakers and service providers in health, social care and housing, especially in rural and coastal areas to enhance service accessibility through developing mobile and outreach services following models that have shown success in reaching homeless populations in dispersed communities.^48^ To address the challenges posed by geographical isolation and limited transportation options, better referral pathways between emergency care services and community support services are needed, with dedicated staff to facilitate these connections. Collaboration among services is essential for integrated care, reducing reliance on emergency services and interventions. This could be achieved through establishing multiagency coordination hubs in key locations, with satellite services reaching into more remote areas, including colocated services where possible and virtual coordination where not. Additionally, incorporating trauma-informed approaches is crucial, given the high trauma incidence, requiring effective and sensitive support. This involves staff training and service design that considers the impact of repeated service transitions in rural and coastal settings. Future research is needed to evaluate the effectiveness of these interventions, particularly examining how mobile and outreach services can best serve dispersed rural and coastal populations. Research is also needed to understand how digital solutions might complement in-person services while accounting for digital access barriers in rural and coastal areas.

## Supplementary material

10.1136/bmjph-2024-001468 online supplemental file 1

10.1136/bmjph-2024-001468 online supplemental file 2

10.1136/bmjph-2024-001468 online supplemental file 3

10.1136/bmjph-2024-001468 online supplemental file 4

10.1136/bmjph-2024-001468 online supplemental figure 1

## Data Availability

Data are available on reasonable request.
