# Supplementary material for: ‘The lights are on, and the doors are always open’: a qualitative study to understand challenges underlying the need for emergency care in people experiencing homelessness in rural and coastal North East England
Source: BMJ Public Health. 2025 Feb 20;3(1):e001468. doi: 10.1136/bmjph-2024-001468 (PMC11842980; doi:10.1136/bmjph-2024-001468)
Supplement: online supplemental file 1 [file bmjph-3-1-s001.pdf]

1 - When completed: 1 for participant; 1 for researcher site file

Baddiley-Clark Building, Newcastle
University, Newcastle upon Tyne,
NE2 4AX

IRAS ID: 311529

Participant Identification Number:

CONSENT FORM

Title of Project: Identifying Multi-Agency, Trauma-Informed, and Integrated Solutions for the Unmet Needs of People Experiencing Homelessness in Northumberland and North Tyneside

If you agree, please initial box

1. *I confirm that I have read the information sheet dated 17/06/2022 version 1.2 for the above study. I have had the opportunity to consider the information, ask questions and have had these answered satisfactorily.*	☐
2. *I understand that my participation is **voluntary** and that I am free to withdraw at any time without giving any reason, without my medical care or legal rights being affected.*	☐
3. *I understand my **[interview/focus group/workshop]** will be audio-recorded and transcribed.*	☐
4. *I understand that the information collected will be used in future reports, articles or presentations by the research team.*	☐
5. *I understand that the information collected about me will be used to support other research in the future and may be shared anonymously with other researchers.*	☐
6. *I understand that any data created from this study will be held in a **[password-protected folder/locked filing cabinet]** for ten years after which the data will be destroyed. All the data collected will be kept anonymous and confidential, and only members of the research team will have access to this data.*	☐
7. *I agree to take part in the above study.*	☐

Name of Participant **Date** **Signature**

Name of Interviewer **Date** **Signature**
