# Supplementary material for: ‘The lights are on, and the doors are always open’: a qualitative study to understand challenges underlying the need for emergency care in people experiencing homelessness in rural and coastal North East England
Source: BMJ Public Health. 2025 Feb 20;3(1):e001468. doi: 10.1136/bmjph-2024-001468 (PMC11842980; doi:10.1136/bmjph-2024-001468)
Supplement: online supplemental file 2 [file bmjph-3-1-s002.pdf]

Baddiley-Clark Building, Newcastle
University, Newcastle upon Tyne,
NE2 4AX

PARTICIPANT INFORMATION SHEET

Title of Project: Identifying Multi-Agency, Trauma-Informed, and Integrated Solutions for the Unmet Needs of People Experiencing Homelessness in Northumberland and North Tyneside

We'd like to invite you to take part in our research study. Before you decide, it is important that you understand why the research is being done and what it would involve for you. Please take time to read this information sheet and discuss it with others if you wish. If there is anything that is not clear, or if you would like more information, please ask us.

What is the purpose of the study?

The purpose of this study is to understand how health and social care organisations in Northumberland and North Tyneside can work together to improve the short, medium and long-term social and health outcomes for people experiencing homelessness. People experiencing homelessness face stigma and barriers to accessing services and specialist support they need. Due to these barriers, people experiencing homelessness are more likely to attend emergency departments (ED). We know a lack of flexibility in services can prevent timely support and make accessing support difficult.

In our study, we would like to speak to people involved in providing support and people who have faced these issues to understand how better care and support can be offered.

Why have I been invited?

You have been invited to take part as you are either:

1. A person who has experienced homelessness or is currently experiencing homelessness and has or would have liked to attend(ed) the emergency department (ED) within Northumberland and North Tyneside.
2. A person who provides care and support to people experiencing homelessness within Northumberland and North Tyneside.

Do I have to take part?

No. Taking part in any part of this study is entirely voluntary. You are able to withdraw if you change your mind at any part of the study, without giving a reason.

What does taking part involve?

There are five parts to this study. You could be asked to participate in either a survey, interview, focus group or workshop. This could involve talking to us for about an hour and our conversation will be audio recorded. Surveys could involve completing a form in-person or online. We would like to talk to you in-person or face-to-face, and we will adhere to social distancing guidelines, or we can also speak to you via telephone or by video call such as Zoom.

Baddiley-Clark Building, Newcastle
University, Newcastle upon Tyne,
NE2 4AX

What are the possible benefits of taking part?

By taking part, you are given an opportunity to share your experiences and opinions and have your views heard. This can contribute towards creating evidence for change and improving services. Your participation can help to increase the quality, availability, and access to appropriate support for similar populations in the future. As a thank you for your time, you might be eligible to receive a £20 voucher, and if needed, we can refund costs for travel or phone credit.

Are there any possible disadvantages or risks from taking part?

Risks are not anticipated with participation in this study. We will ask you to share your personal experiences and we recognise that you may share experiences that might have been negative or traumatic. If this happens, you can pause or stop at any point. There are resources you can access or organisations you can speak to if you do feel like you need additional support. We can provide information on some of these at any point during our conversations and will provide you with a sheet at the end of our conversation with this information.

How will we use information about you?

We will need to use information from you for this research project. All the information that we collect about you for this study will be kept safe, secure, and strictly confidential, and will be respected subject to legal constraints and professional guidelines. We will use your name and contact details (e.g., telephone number, email address) only to contact you about the research study. Only our study team will have this information. We will use these details in order to process and to check the accuracy of the research study. People who do not need to know who you are will not be able to see your name or contact details. Your data will have a code number instead. Once we have finished the study, we will keep some of the data so we can check the results. We will write our reports and publications in a way that no-one can work out that you took part in the study.

As part of this study, interviews, focus groups and workshops will be audio-recorded and typed up word for word. Any information that could possibly identify you will be removed so that this information is fully anonymous. Anonymised quotes from these conversations will be used in study reports, publications and for educational purposes. Your anonymised data will become part of a dataset which can be accessed by researchers at Newcastle University and only for research purposes. Making sure we keep everything you share with us private is really important. When it comes to keeping what you tell us private, we will take a similar approach to a nurse or a doctor. This means that everything you say will be kept private, unless you share something that makes us worried about your or someone else's safety. If information is shared that indicates significant risks to themselves or others then confidentiality will be breached and Chief Investigator (Sheena Ramsay) will be informed in the first instance, and if deemed appropriate further action taken in line with Newcastle University safeguarding policies and procedures. Findings from this study will be shared in academic journals or at conferences, and possibly used to inform other studies.

If you decide to withdraw at any point during the study, identifiable data already collected with your consent would be retained and used in the study. No further data would be collected, or any other research procedures carried out on or in relation your data. The information you provide will be stored securely on a password protected Newcastle University network. All data will be stored in accordance with university guidelines and data laws (GDPR). Personal identifiable information, such as full name, contact telephone details or email addresses will be destroyed when the study ends.

Baddiley-Clark Building, Newcastle
University, Newcastle upon Tyne,
NE2 4AX

What are your choices about how your information is used?

You can stop being part of the study at any time, without giving a reason, but we will keep information about you that we already have. We need to manage your records in specific ways for the research to be reliable. This means that we won't be able to let you see or change the data we hold about you. If you agree to take part in this study, you will have the option to take part in future research using your data saved from this study.

Who is funding this research?

This study is funded by the National Institute for Health Research: Research for Social Care: Mental health research in Northern England - Round 1.

Has this study received ethical approval?

All research in the NHS is looked at by an independent group of people, called a Research Ethics Committee. This study has been reviewed and approved by a local Research Ethics Committee, who ensure that you are protected in terms of your health and your rights. This study has also been reviewed by the Health Research Authority and a Confidentiality Advisory Group. Elements of this study are also reviewed by Newcastle University and Changing Lives' Ethics Committees.

Who is the sponsor and data controller for this research?

Newcastle University is the sponsor for this study based in the United Kingdom. Newcastle University will be analysing your data in order to undertake this study and will act as the data controller for this study. This means that Newcastle University is responsible for looking after your information and using it properly.

The lawful basis for carrying out this study under GDPR is Task in the Public Interest, (Article 6,1e) as research is cited as part of the University's duties. Your rights to access, change or move your information are limited, as Newcastle University need to manage your information in specific ways in order for the research to be reliable and accurate. If you withdraw from the study, Newcastle University will keep the information about you that has already been obtained. To safeguard your rights, the minimum personally-identifiable information will be used. You can find out more about how Newcastle University uses your information by contacting their Data Protection Officer [Maureen Wilkinson, rec-man@ncl.ac.uk].

Who should I contact for further information relating to the research?

You can contact us by email on [insert email] or by phone on [insert telephone number].

Population Health Sciences Institute, Newcastle University, The Baddiley-Clark Building, Richardson Road, Newcastle upon Tyne NE2 4AX.

What if there is a problem?

Sheena Ramsay, Clinical Senior Lecturer & Hon Consultant in Public Health, Population Health Sciences Institute, Newcastle University, The Baddiley-Clark Building, Richardson Road, Newcastle upon Tyne NE2 4AX: Sheena.Ramsay@newcastle.ac.uk

Baddiley-Clark Building, Newcastle
University, Newcastle upon Tyne,
NE2 4AX

Where can you find out more about how your information is used?

You can find out more about how we use your information:

- at www.hra.nhs.uk/information-about-patients/
- our leaflet available from www.hra.nhs.uk/patientdataandresearch
- by asking a member of the research team
- by sending an email to steven.thirkle@newcastle.ac.uk

Resources

If you are affected by any of the issues raised in this research please visit:

- **NHS advice:** https://www.nhs.uk/conditions/coronavirus-covid-19/symptoms/
- **NHS – Where to get urgent help for mental health:** https://www.nhs.uk/using-the-nhs/nhs-services/mental-health-services/where-to-get-urgent-help-for-mental-health/
- **Victim Support – support for people affected by crime or traumatic events, including hate crime:** https://www.victimsupport.org.uk/
- **Samaritans – Emotional support for everyone:** www.samaritans.org Samaritan provides confidential non-judgmental emotional support, 24 hours a day. The Samaritans can be called on 116 123.
- **Mind – Advice and support for anyone with a mental health problem:** www.mind.org.uk. The line is open from 9am-6pm, Monday to Friday (except for bank holidays). The infoline can be reached on 0300 123 3393. You can also text Mind at 86463.**SANE** runs a national, out-of-hours mental health helpline offering specialist emotional support and information to anyone affected by mental illness, including family, friends and carers. The helpline is open every day of the year from 6pm to 11pm. The helpline can be reached on 0300 304 7000
- **Anxiety UK** helpline on 03444 775 774, open Mon-Fri 09:30-22:00 and Sat-Sun 10:00-20:00

Thank you for reading this information sheet and considering taking part in this research.

Baddiley-Clark Building, Newcastle
University, Newcastle upon Tyne,
NE2 4AX
