# Supplementary material for: ‘The lights are on, and the doors are always open’: a qualitative study to understand challenges underlying the need for emergency care in people experiencing homelessness in rural and coastal North East England
Source: BMJ Public Health. 2025 Feb 20;3(1):e001468. doi: 10.1136/bmjph-2024-001468 (PMC11842980; doi:10.1136/bmjph-2024-001468)
Supplement: online supplemental file 3 [file bmjph-3-1-s003.pdf]

INTERVIEW/FOCUS GROUP TOPIC GUIDE

Topic guide for people with experience of homelessness

Title of Project: Identifying Multi-Agency, Trauma-Informed, and Integrated Solutions for the Unmet Needs of People Experiencing Homelessness in Northumberland and North Tyneside

Interviewer Notes

The interview/focus group focuses on understanding the underlying needs of people with lived experiences of homelessness and why people experiencing homelessness are accessing emergency healthcare services and whether their needs could be better supported elsewhere.

The high-level questions under investigation are:

- The reasons underlying ED attendance and experience of attendance
- Underlying health (including mental health) needs
- Underlying social care needs
- Awareness of other support services
- Access to other support services
  - Was there any other contact with health services? (I.e., GPs)
  - Was there any contact with social care services?
  - Was there any contact with statutory services?
- What could have been done before ED attendance?
- What needs to have happened after ED discharge?

1. Introduction

- Introduce yourself and thank them for agreeing to talk to us.
- Explain that the conversation will last about an hour and that the participant can stop at any point.
- *I would like to tell you about the study and what's involved so that you are able to decide whether you wish to proceed further* (recap information sheet and section 3).
  - Double-check eligibility criteria
    - Aged 18 and above
    - Accessed any type of emergency service for health reasons
    - Experience of homelessness
- Complete consent forms and demographic characteristics form
- *Participation is voluntary and you can stop the conversation or decline to answer specific questions at any time should you wish.*

2. Confidentiality and Consent

- Reiterate issues of confidentiality and anonymity, the purpose of the study and that the data will only be used for research purposes.
- **Verbally confirm that participants understand the purpose and confidentiality of the research, that they are happy to take part and that the session will be audio recorded**
- Ask if the participant has any questions

3. What is this Research About?

- *Can I start by telling you about this research study? People who experience homelessness sometimes reach out to hospital A&E or other emergency health services (such as ambulance or urgent care). We want to understand what led to needing emergency care and find out what can be done to avoid this situation arising in the first place – and if they could be better supported somewhere else. This information will help us find out what other support might be needed.*
- *The first step is to understand the issues people experiencing homelessness face who may have had emergency services presentations (such as the emergency department, ambulance services, and urgent care). We want to hear from people with experience of these issues – and that's what our conversation today is about.*

4. Interview Script

Experience Accessing Emergency Services

Interviewer Notes

In the first part of the interview, we want to understand why the participant needed emergency health care and what was involved in accessing that emergency service.

- Why did they reach out to emergency service
- Which service they have reached out to
- How they accessed the service
- What happened during their visit; what kind of support did they get; where did they go
- What happened when they were discharged
- Previous attendance at emergency services
- What needs to happen when people experiencing similar challenges are discharged
- Awareness of other support services
- Access to other support services

Thank you for agreeing to speak with me today. We are trying to understand why people experiencing homelessness need to reach out to emergency services and so I would like to begin by talking about why you accessed emergency services if that's ok?

1. ***Can you tell me why you needed to reach out to emergency health services?*** (Note: why they needed to reach out or what led them to it.)

Example: what led you to reach out?

2. ***Where did you go? And how did you reach out to that service?*** (Note: where did they go (e.g. ED, ambulance, walk-in) and how did they access that service.)
   Example: how did you find out about the service, or who helped you access that service? where did you have to go to reach the service? was it an accident & emergency department, did you call 999 or 111, or was it an urgent treatment centre or minor injury unit?

3. ***Can you tell me what happened during that visit?*** (Note: what happened during their visit; what kind of support did they get?)
   *3.1. Probe: **whom** did you see?*
   Example: did you see a nurse, a doctor, or somebody else?
   *3.2. Probe: **how** did they help you?*
   *3.3. Probe: **did** you feel like you got the help you needed?*

*3.3.1. If yes to Q3.3, probe further: **how** did it help you? what was good about it?*
*3.3.2. If no to Q3.3, probe further: **what** was not good about it?*

*4. **What happened at the end of that visit or when you finished with the emergency service? Were you referred to some other support?*** (Note: what happened when they were discharged)

Example: where did you go after your visit?

*4.1. Probe: **where** did you go?*
*4.2. Probe: **was** it helpful?*
*4.3. Probe: **would** anything else have helped?*

5. ***What do you think needs to happen after people experiencing homelessness are sent on from emergency services?*** (Note: what needs to happen when people experiencing similar challenges are discharged)

*6. **Have you been in a similar situation before needing emergency care?*** (Note: previous attendance)

Example: have you accessed emergency services before?

*6.1. If yes to Q6, probe: **can** you tell me more about that situation? **Was** it for something similar?*
*6.2. Probe: **what** help did you get at that time?*
*6.3. Probe: **whom** did you see?*
*6.4. Probe: **where** did you go?*

7. ***What could have helped earlier or before you needed to reach out to emergency services?*** (Note: what could have been done before ED attendance?)

Example: looking back at your past experience, could anything have helped you before?

8. ***Did you get help from any other health service before reaching out to emergency services?*** (Note: contact with any other support)

Example: did you access other support services? other support may include accessing food banks, charities, hostels or housing providers.

*8.1. If yes to Q9, probe: **can** you tell me more about your contact with other support services?*
*8.2. Probe: **was** there any other contact with health (I.e., GPs), social care or statutory services?*

Thank you for sharing your experience in accessing emergency services. I'd like to chat about other support and services that are available in the region if that's ok?

*9. **In your experience, what has accessing support outside of emergency services been like?*** (Note: access to other support)

Example: do you know who to reach out to for help and support for issues that you are facing?

9.1. Probe: do you feel you know where to get support and help, or whom to contact?
*9.2. Probe: **would** these services have been able to offer you more help?*
*9.2.1. If yes, probe further: **how** would these have been able to help you?*
*9.2.1.1. Probe further: if these services can offer you more help, **may** I ask why you didn't choose to access this service first?*
*9.2.2. If no, probe further: **why** are these services not able to offer you the help you need?*

*10. **Emergency services may not always be the best place for help with some of these issues. Where do you think people experiencing homelessness are finding out about the support available?*** (Note: where are people experiencing homelessness discovering routes into support?)

Health Needs

Interviewer Notes
In this part of the interview, we want to understand the existing health challenges that the participant is facing or has faced in the past, whether they are receiving support – and what that support is.<br>• Physical or mental health conditions<br>• Treatment or support for any of these conditions<br>• Traumatic experiences

11. ***Can we talk a bit about the health issues that may have led to you needing emergency care - are there any particular health issues, which may have led to you needing emergency care?*** (Note: underlying health conditions)

Example: this could be a physical condition such as pain or being unable to walk very far, it could be a diagnosis by a doctor such as diabetes or high blood pressure. It could also be a mental health condition such as anxiety, depression, or any other diagnosis by a doctor.

12. ***Have you received help for these health issues?*** (Note: help and support for these conditions)

Example: have you spoken to a nurse or a doctor or are you seeing a healthcare professional for these issues?

- 12.1. *If yes, probe: **what** sort of help are you receiving? Can you tell me more about **how** you got this help?*
  - 12.1.1. *Probe: **who** did you go to first for help?*
  - 12.1.2. *Probe: **did** you know where to go?*
    - 12.1.2.1. *If yes, probe: **how** did you find out about the support and **where** to go?*
  - 12.1.3. *Probe: **what** kind of help or support did you get?*
  - 12.1.4. *Probe: **did** you get any other help or support or treatment?*
    - 12.1.4.1. *If yes, probe: **where** did you go? **Whom** did you see? **What** was involved in that?*
- 12.2. *If no, probe: **have** you tried to get any help?*
  - 12.2.1. *If no, probe: **why** is that? **what** has stopped you from getting help? **What** will help you reach out for help?*
- 12.3. *If yes, probe: so, **did** you try, and not get any help? **Why** was that? Can you tell me a bit more about **what** happened?*

13. ***Did anything happen in the past which may have led to these health issues or made them worse?*** (Note: identifying health decline)

Example: do you know what happened that caused these health issues?

- 13.1. *Probe: do you think it was something that could have been prevented?*

14. ***Sometimes people can be affected by negative experiences or stresses – has that been the case for you? (If so, have those negative experience or stresses changed the way you seek help?)*** (Note: traumatic experiences)

Example: negative or stressful experiences shape how we perceive care and people may find services difficult to access going forward.

Social Needs - Underlying Need for Emergency Care

Interviewer Notes
In this part of the interview, we want to understand the existing social care challenges that the participant is facing (particularly underlying need for emergency care) and whether they are receiving support.<br>• Social conditions<br>• Support for any of these conditions

15. ***We have just spoken a lot about health issues, and we know there are things other than health, which could have led to needing emergency care. Some of these issues could be challenges with looking for jobs, or housing, or day-to-day tasks such as budgeting. Do you find you are facing any of these issues or anything similar?*** (Note: underlying social conditions)

Example: can you tell me a bit more about the other issues you are finding challenging?

15.1. *Probe: **what** do you find difficult about [repeat participant's challenge]?*

15.2. *Probe: w**hen** did this start?*

15.3. *Probe further: Social challenges can be even harder for people with disabilities. **Do** you face similar challenges?*

16. ***Have you had any help with any of these issues?*** (Note: help and support for these conditions)

Example: this could be a support worker, family or friends that help you.

16.1.1. *Probe: **who** did you see?*

Example: did you see a support worker, or somebody else?

16.1.2. *Probe: **how** did they help you?*

16.1.3. *Probe: **did** you feel like you got the help you needed?*

16.1.4. *Probe further: **how** did it help you? What was good about it?*

16.1.5. *Probe further: **what** was not good about it?*

16.2. *If no to Q18, probe further: is it something that you would like help with?*

16.2.1. *Probe further: **why** would you not like help with this?*

16.2.1.1. *Probe further: **what** needs to change to make you want to seek help?*

Final Questions

17. ***Is there anything service providers should be aware of when trying to support people experiencing homelessness?*** (Note: traumatic experiences)

Example: Services might be more inclusive by being aware that people experiencing homelessness may have had a traumatic experience with the service.

17.1.1. *Probe: **what** else do you think is needed?*

18. ***Thank you very much for your time today and for sharing your experiences with me. Before we wrap up, is there anything you feel we have missed in our conversation that you would like to mention?***

Reiterate the study aim and objectives and provide a copy of the debrief sheet.

5. Appendices

1. Simplified informed consent for remote interviews also to use in in-person interviews to help talk through the questions but need to ask participant to initial each question on consent form.

I'd like to go through a few questions just to make sure that you are happy to take part in the study, and how we collect your information for research.

1. First, do you agree that you've read and understood our information sheet and you've had the chance to ask us questions?

2. Do you agree that your taking part is voluntary and you are free to withdraw any time without giving a reason?

3. Our chat is being audio recorded. The recordings will be stored securely without your name and any other personal details and will only be used for research purposes only for this study and further research. We will have your name and contact details only for contacting you for this study. Is that ok with you?

4. The information we talk about today (or data) will be used as part of our research and will later be published as a report, without any names or personal details. Are you happy with that?

5. So, are you happy to take part in this research study?
