# Supplementary material for: ‘The lights are on, and the doors are always open’: a qualitative study to understand challenges underlying the need for emergency care in people experiencing homelessness in rural and coastal North East England
Source: BMJ Public Health. 2025 Feb 20;3(1):e001468. doi: 10.1136/bmjph-2024-001468 (PMC11842980; doi:10.1136/bmjph-2024-001468)
Supplement: online supplemental figure 1 [file bmjph-3-1-s005.pdf]

Work Package 1B – Coding Framework for Interviews and Focus Groups

Research objectives: To investigate the broad range of factors leading to people experiencing homelessness frequently presenting at emergency department (ED). A qualitative approach will allow us to understand how the needs of this population can be better addressed, and what support can be provided to people experiencing homelessness, so that they receive appropriate support.

Method:

To gain insights from people who have experienced homelessness and have accessed ED, and also from service providers in relevant health and social care organisations.

Strategy:

Deductive coding using initial research topics

1. Emergency care presentation
2. Health issues
3. Social issues
4. Trauma

Structure of interview questions to structure categories into hierarchical coding frame (theme – sub-theme). Codes developed from initially coding five transcripts.

Table 1 - Coding Framework

Code level one (Interview Topics)	Code level two (Initial codes)	Initial theme development - sub-themes	Final Themes and sub-themes	Keywords
Emergency care presentation	• Lack of routes for help<br>• Distrust in services<br>• Geographic isolation<br>• Limited resources<br>• Lack of digital access<br>• Transportation issues<br>• Administrative barriers	Difficult to find and reach out to appropriate support when managing difficult situations<br><br>The total absence of a route for help<br><br>Low (and often traumatic) expectations from services due to negative past experiences	**Accessibility Challenges in Rural and Coastal Settings**	• Providing timely help<br>• Helping people reach out<br>• Not worthy of help<br>• Complicated pathways for help
Health issues	• Poor physical health<br>• Poor mental health<br>• Substance use<br>• Disabilities<br>• Unhelpful support<br>• Geographic isolation<br>• Limited resources<br>• Lack of digital access<br>• Transportation issues<br>• Administrative barriers	Harmful situations (rough sleeping) contribute to poor physical health which lead people to coping with substances which then lead to poor mental health<br><br>Disabilities such as dyslexia, ADHD and other learning difficulties mean traditional methods of accessing support are not helpful<br><br>Unexplored disabilities which may prevent people from being able to use systems as intended	**Fragmented Support in Rural and Coastal Settings**	• No treatment<br>• Using substances to help manage conditions
Social issues	• Poor personal and social education	Histories are often associated with turbulent childhoods and	**Service Restrictions and Limited Alternatives**	• Preference to sleep rough<br>• Housing criteria

	• Negative social environments<br>• Stigma<br>• Social vulnerabilities<br>• Rigid eligibility requirements<br>• Failure to accommodate complex needs<br>• Lack of trauma-informed approaches	family lives and good education was not available<br><br>Current social environments are unhelpful to recovery from substance use and trauma		• Complicated pathways for help
Trauma	• Adverse Experiences<br>• Insensitive/triggering services<br>• Rigid eligibility requirements<br>• Failure to<br>• accommodate complex needs<br>• Lack of trauma-informed approaches	Adverse childhoods and difficult past and current experiences are common and impact people – often these are untreated<br><br>Distrust and lack of safety in services can drive people back to the street where they feel safer – rather than services helping people, people are feeling harmed further by them	**Prioritisation of Needs**	• Preference to sleep rough
Miscellaneous	• Lack of communication between services<br>• Staff related issues - lack of training and understanding	Services are not able to connect (long-term) due to short life spans of VCSE organisations<br><br>Staff often recognise they are not equipped with the skills to fully support their client and so “pass the duty on” – unable to support multiple needs	**Services are working in silos and the front-line services that are able to help do not have the appropriate training to do so**	• Staff do not have skills required<br>• Lack of understanding by staff in services<br>• Staff are overworked<br>• Working together (multi-agency)
